# Strong Correlation of Renal Function with Choroidal Thickness in Patients with Type 2 Diabetes: Retrospective Cross-Sectional Study

**DOI:** 10.3390/jcm9072171

**Published:** 2020-07-09

**Authors:** Min Gyu Choi, Jee Taek Kim

**Affiliations:** 1 Department of Ophthalmology, College of Medicine, Chung-Ang University Hospital, Seoul 06973, Korea; choimg7@naver.com; 2 510 Air Defense Artillery Battery, 1st Air Defense Missile Brigade, Air Defense & Guided Missile Command, Republic of Korea Air Force, Pohang 37928, Korea; 3 Biomedical Research Institute, Chung-Ang University Hospital, Seoul 06973, Korea

**Keywords:** diabetes complications, diabetes mellitus, diabetic retinopathy, diabetic nephropathies, choroidal thickness, optical coherence tomography

## Abstract

The purpose of this study was to analyze the correlation between renal function and subfoveal choroidal thickness (SFChT) in treatment-naïve proliferative diabetic retinopathy (PDR) patients. This study included 85 eyes of 52 treatment-naïve PDR patients who underwent kidney function testing and urinalysis and 42 eyes of 33 age-matched controls. Treatment-naïve eyes with PDR were categorized into pachychoroid and leptochoroid groups based on the SFChT of the control group. Kidney function profiles were compared between pachychoroid and leptochoroid groups; the relationship between kidney function profile and SFChT was evaluated using regression analysis. Compared with the pachychoroid group, the leptochoroid group had significantly higher serum creatinine (*p* = 0.026), cystatin C (*p* = 0.004), and phosphorus (*p* < 0.001) levels and a lower estimated glomerular filtration rate (eGFR) (*p* < 0.001). Multivariate linear regression analyses showed that SFChT was positively correlated with eGFR (Cystatin C) (*p* = 0.007) and negatively correlated with serum phosphorus (*p* = 0.001). SFChT of patients with eGFR < 30 mL/min/1.73 m^2^ and serum phosphorus level ≥4.0 mg/dL was less than that of patients with higher eGFR and lower serum phosphorus level. The choroidal thickness of treatment-naïve PDR patients is closely affected by renal function. Kidney function test should be considered if SFChT of patients with treatment-naïve PDR is reduced.

## 1. Introduction

Diabetic retinopathy (DR) is a major cause of visual deterioration worldwide and is estimated to affect up to 35% of diabetes patients [1]. The choroid is a highly vascularized layer that surrounds and supports the retina, [2] and it plays an important role in the pathogenesis of DR; moreover, abnormalities in choroidal vasculature may be responsible for visual impairment in eyes with DR [3]. Furthermore, several studies have described the presence of diabetic choroidopathy in the eyes of patients with DR [4,5].

With the recent evolution of optical coherence tomography (OCT) techniques, such as enhanced depth imaging OCT and swept-source OCT (SS-OCT), high-resolution scans of the retina and choroid can be obtained in a non-invasive manner. Moreover, SS-OCT uses longer wavelengths and faster scanning speed lasers, which allow for deep range imaging and increased averaging [6]. Using these techniques, several studies have revealed the relationship between choroidal thickness (ChT) and various systemic factors such as age, sex, and hormonal change or diseases, including systemic hypertension or cardiovascular disease, systemic inflammatory conditions, hematological diseases, and neurological diseases [7]. The choroid can be affected by these systemic conditions because it is a highly vascular tissue with the highest level of blood flow in the body [8].

Diabetes mellitus (DM) is the leading cause of chronic kidney disease (CKD) and end-stage renal disease (ESRD) [9]. The 20-year risk of diabetic nephropathy is 41% in type 2 DM patients who were initially free of proteinuria [10]. When proteinuria occurs, the subsequent 10-year risk for progressive CKD is 11% [11]. However, the effect of CKD on the choroid of DM patients has not really been systematically investigated until now. Only a few studies have been performed to reveal the effects of microalbuminuria in CKD on the choroid [12,13]. However, the findings of these studies are inconsistent [12,13]. Therefore, this study aimed to systematically analyze the correlation between renal function and SFChT in treatment-naïve proliferative diabetic retinopathy (treatment-naïve PDR) patients.

## 2. Materials and Methods

### 2.1. Study Design

This study was a retrospective, cross-sectional, and observational study. This research adhered to the tenets of the Declaration of Helsinki and was approved by the Institutional Review Board committee of the Chung-Ang University Hospital, Seoul, South Korea. Informed consent was waived by the approving IRB because of the retrospective nature of the study (IRB No. 1701-001-16025).

### 2.2. Study Subjects

Medical records of patients with type 2 diabetes who visited the retina clinics of Chung-Ang University Hospital from 1 September 2016 to 30 September 2019 were retrospectively reviewed. Only treatment-naïve eyes with PDR were included. All patients underwent comprehensive ocular examinations, fundus examination, photography, and SS-OCT (DRI Triton™; Topcon, Tokyo, Japan). The DM control state was evaluated from venous blood by measuring fasting blood sugar (FBS) and glycated hemoglobin (HbA1c) levels and calculating the recent difference in HbA1c over the last 3 months (ΔHbA1c). Renal function was evaluated based on the following parameters: serum creatinine, cystatin C, phosphorus, calcium levels, and estimated glomerular filtration rate (eGFR) using cystatin C. Urine samples were obtained for routine urinalysis and measurement of albumin level, creatinine level, and albumin/creatinine ratio (ACR). The mean arterial pressure was calculated using the following equation: diastolic pressure + 1/3(systolic pressure—diastolic pressure). Body height and weight were recorded to calculate body mass index (BMI). Patients whose laboratory workups were performed within a span of 4 weeks from ophthalmic evaluations were selected.

Exclusion criteria were refractive error ≥3.0 diopters spherical equivalent, diagnosis of ocular hypertension or glaucoma with an optic nerve cup/disk ratio >0.6, prior cataract or retinal surgery, any treatment history (including panretinal photocoagulation (PRP) or intravitreal injection), history of ocular trauma, diagnosis of any eye disease including retinal and choroidal diseases, and systemic disease other than DM or hypertension that may affect ChT. Eyes with low-quality OCT images (image quality index <60) or media opacities, such as vitreous hemorrhage and cataract, were also excluded.

### 2.3. Identification of PDR and DME

The severity of DR was graded according to the Early Treatment Diabetic Retinopathy Study (ETDRS) retinopathy severity scale. Fluorescein angiography was performed using an ultrawide-field confocal scanning laser ophthalmoscope (Optos Panoramic 200MA™; Optos PLC, Dunfermline, Scotland, UK) to identify new vessels on the disc or elsewhere on the fundus. PDR was defined as eyes with new vessels on the disc or elsewhere on the fundus with the presence of prominent focal leakage from the new vessels. Slit-lamp examination and SS-OCT was performed for diagnosing diabetic macular edema (DME).

### 2.4. Imaging Protocol

**Figure 1:**
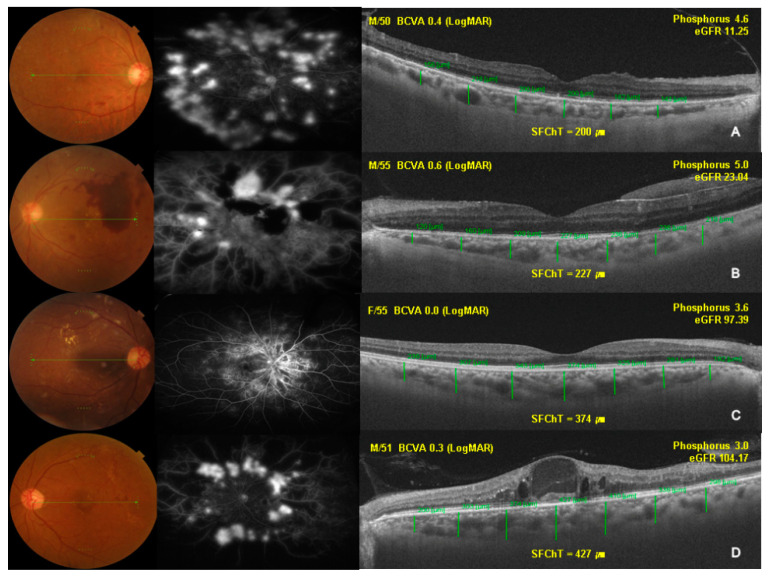
Representative cases showing choroidal thinning and thickening in treatment-naïve eyes of patients with proliferative diabetic retinopathy (PDR). Patients with thinner choroids (**A**,**B**) exhibited a more advanced stage of chronic kidney disease (CKD) than those with thicker choroids (**C**,**D**). BCVA, best-corrected visual acuity; LogMAR, logarithm of the minimum angle of resolution; eGFR, estimated glomerular filtration rate.

SS-OCT was performed using a wavelength of 1050 nm and a scan speed of 100,000 amplitude scans/s, which yielded an axial resolution of 8 μm and a depth of 2.4 dB/mm; OCT B-scan imaging was performed using a 6 × 6 mm three-dimensional cube-scan and a 9 mm five-line cross-scan (measuring time: 10:00 AM to 16:00 PM). Central retinal thickness (CRT) values were obtained from automatic ETDRS grid maps in the 6 × 6-mm three-dimensional cube-scan mode after confirming the position of the grid. The mean retinal thickness (RT) was measured at eight locations (except at the central fovea) on the automatic ETDRS grid map, and the average of the measured RT values was calculated. ChT was measured at seven locations (in the subfoveal region and at 1000 μm intervals from the fovea to the 3000 μm mark in the nasal and temporal positions) using the 9 mm five-line cross-scan with a built-in caliper tool, as was the distance between the Bruch’s membrane and the choroid-sclera interface (Figure 1). The mean ChT was the average of the ChT values at the seven locations.

Two independent observers (M.G.C. and J.T.K.), who were blinded to the clinical data of each patient, measured the ChT of each eye, and the average value was used in data analysis to avoid interobserver variation. When SFChT was measured in both eyes, the mean value of both eyes was used. Control data were obtained from the eyes of age-matched patients who visited the retina clinic for treating idiopathic epiretinal membrane or macular hole.

### 2.5. Main Outcome Measure

Using the mean SFChT value of healthy controls (261.5 ± 21.2 μm) as a cut-off value, the patients were divided into two groups based on the mean SFChT value of both eyes of each patient; the groups were as follows: the pachychoroid group (thick choroid group) and the leptochoroid group (thin choroid group).

Clinical characteristics and renal function parameters including serum creatinine, cystatin C, phosphorus, calcium levels, eGFR, urine albumin, and urine ACR were compared between pachychoroid and leptochoroid groups; the relationship between these profiles and SFChT was evaluated using regression analysis. SFChT was compared according to the severity of eGFR, urine ACR, and serum level of phosphorus; SFChT was also compared between patients who underwent renal replacement therapy (dialysis) and those of patients who did not.

### 2.6. Categorization of Patients according to eGFR and ACR

Patients were categorized into eGFR < 30 mL/min/1.73 m^2^ (severely decreased), eGFR with 30–60 mL/min/1.73 m^2^ (mildly to moderately or moderately to severely decreased), and eGFR ≥ 60 mL/min/1.73 m^2^ (normal or mildly decreased), and were categorized into ACR < 30 mg/g (normal to mildly increased), ACR with 30–300 mg/g (moderately increased), and ACR ≥ 300 mg/g (severely increased), according to the criteria of the Kidney Disease: Improving Global Outcomes (KDIGO) CKD Work Group [14].

### 2.7. Statistical Analyses

Statistical analyses were performed using SPSS software (version 25.0; IBM Corp., Armonk, NY, USA). The obtained data were analyzed using the chi-squared test, independent *t*-test, paired *t*-test (comparison of two groups), Pearson correlation analysis, and one-way analysis of variance with post-hoc analysis. To reveal the correlation between renal function and SFChT, univariate and multivariate regression analyses and generalized linear model analysis between SFChT and kidney biomarkers were performed. Statistical significance was defined by a *p*-value < 0.05.

## 3. Results

### 3.1. Baseline Characteristics

In total, 85 eyes of 52 treatment-naïve PDR patients (32 males and 20 females; mean age: 52.9 ± 11.0 years; range: 29–75 years; mean duration of diabetes: 11.5 ± 8.4 years; range: 0.3–34.2 years) and 42 eyes of 33 healthy controls (16 males and 17 females; mean age: 54.9 ± 15.1 years; range: 18–86 years) were enrolled in this study. There were no significant differences in age, BMI, refractive error, and IOP between the two groups. Treatment-naïve PDR patients had a significantly lower mean logarithm of the minimum angle of resolution (LogMAR) best-corrected visual acuity (0.24 ± 0.4, 0.05 ± 0.07, *p* < 0.001) and more edematous features on the retina (CRT, 268.3 ± 84.9 µm; 227.9 ± 26.3 µm; *p* = 0.002) than healthy controls. The interobserver reproducibility of SFChT ranged from 0.985 to 0.991. The baseline data of the study population are listed in Table 1. Four representative cases of choroidal thinning and thickening in eyes with naïve PDR patients are shown in Figure 1.

### 3.2. Comparison of Renal Function Parameters IN Groups Classified according to SFChT

To investigate the correlation between renal function and SFChT, we compared clinical characteristics and systemic profiles between patients with thick choroids and those with thin choroids. The mean SFChT of treatment-naïve PDR patients was 316.0 ± 80.2 µm. The mean SFChT of the pachychoroid group was 362.0 ± 45.8 μm (*n* = 30 patients, 57.7%), whereas that of the leptochoroid group was 236.8 ± 30.8 μm (*n* = 22 patients, 42.3%). A significantly higher prevalence of systemic hypertension was observed in the pachychoroid group than in the leptochoroid group (53.1%; 22.4%, *p* = 0.027). However, there were no significant differences in the mean arterial pressure, age, BMI, DM duration, FBS, HbA1c, and ΔHbA1c values of the two groups. Significantly higher serum creatinine (1.8 ± 1.3 mg/dL; 1.1 ± 0.8 mg/dL, *p* = 0.026), cystatin C (2.2 ± 1.2 mg/L; 1.2 ± 0.7 mg/L, *p* = 0.004), and phosphorus (4.2 ± 0.5 mg/dL; 3.4 ± 0.5 mg/dL, *p* < 0.001) levels, and lower eGFR (45.1 ± 34.4 mL/min/1.73 m^2^; 91.8 ± 37.8 mL/min/1.73 m^2^, *p* < 0.001), were noted in the leptochoroid group than in the pachychoroid group. Serum calcium levels, urine albumin levels, and urine ACR were not significantly different between the two groups (Table 2).

### 3.3. Association between SFChT and Renal Function Parameters in PDR Patients

**Figure 2:**
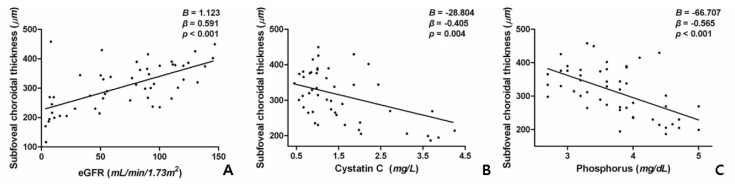
Univariate association between subfoveal choroidal thickness and cystatin C and phosphorus levels and the estimated glomerular filtration rate in patients with proliferative diabetic retinopathy. (**A**). Estimated glomerular filtration rate (eGFR) is positively correlated with subfoveal choroidal thickness (SFChT). (**B,C**). Serum cystatin C (**B**) and phosphorus (**C**) levels are negatively correlated with SFChT.

To investigate which kidney biomarkers most associated with the choroid, we performed univariate and multivariate linear regressions. In the univariate analysis, SFChT was significantly associated with eGFR (*p* < 0.001), cystatin C level (*p* = 0.004), and serum phosphorus level (*p* < 0.001) (Table 3, Figure 2). In the multivariate analysis, greater SFChT was associated with higher eGFR (*p* = 0.009) and lower serum phosphorus levels (*p* = 0.002) (Table 3).

### 3.4. SFChT according to eGFR, Renal Replacement THERAPY, and Urine ACR

**Figure 3:**
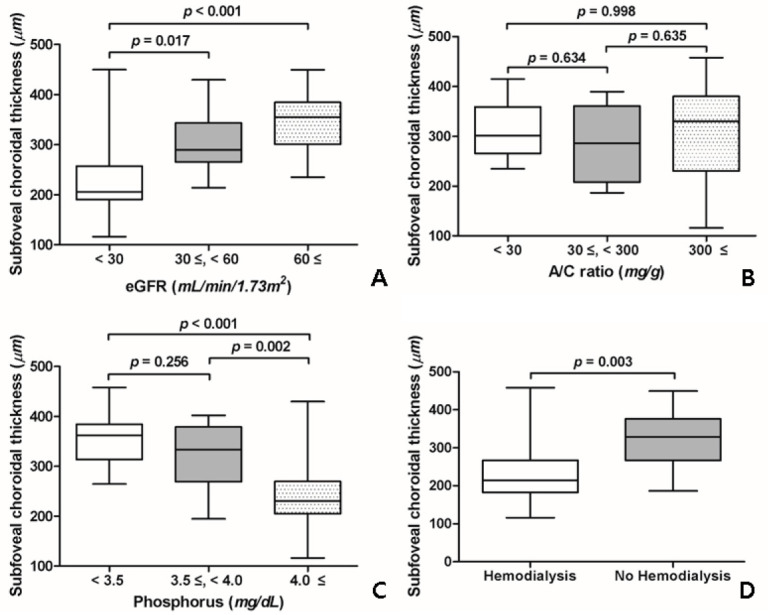
Changes in subfoveal choroidal thickness (SFChT) according to renal function. (**A**) Changes in SFChT according to the estimated glomerular filtration rate (eGFR). Patients with advanced nephropathy tended to have less SFChT. SFChT of patients with eGFR < 30 mL/min/1.73 m^2^ was significantly lesser than that of patients with eGFR ≥60 mL/min/1.73 m^2^ and those with eGFR 30–60 mL/min/1.73 m^2^. (**B**) Changes in SFChT according to the urine albumin/creatinine (A/C) ratio. SFChTs of patients were not significantly different when analyzed according to the severity of urine albumin/creatinine ratio. (**C**) Changes in SFChT according to the serum phosphorus level. Patients with higher serum phosphorus levels had significantly less SFChT than those with lower serum phosphorus levels. (**D**) The difference between SFChT of patients who underwent renal replacement therapy and that of those who did not. Patients who underwent dialysis had significantly less SFChT than those who did not.

To analyze SFChT according to the severity of CKD, we analyzed the changes in SFChT according to the severity of eGFR, urine ACR, and serum level of phosphorus; we also compared SFChT values of patients who underwent renal replacement therapy (dialysis) with those of patients who did not. Patients with eGFR < 30 mL/min/1.73 m^2^ (228.0 ± 80.3 µm) had significantly less SFChT than patients with eGFR ≥60 mL/min/1.73 m^2^ (342.5 ± 57.7 µm, *p* = 0.026) and those with eGFR 30–60 mL/min/1.73 m^2^ (303.4 ± 61.3 µm, *p* < 0.001) (Figure 3A). However, the SFChT of patients with urine ACR ≥300 mg/g was not significantly different from that of patients with urine ACR < 30 mg/g and those with urine ACR 30–300 mg/g (Figure 3B).

Patients with phosphorus level ≥4.0 mg/dL (244.9 ± 75.2 µm) had significantly less SFChT than those with phosphorus level < 3.5 mg/dL (357.8 ± 53.7 µm, *p* < 0.001) and those with phosphorus level 3.5–4.0 mg/dL (322.2 ± 59.1 µm, *p* = 0.002) (Figure 3C). The correlations between renal function parameters were shown in Table S1. Patients who underwent renal replacement therapy (hemodialysis) had significantly less SFChT than those who did not undergo dialysis (236.8 ± 95.9 µm; 320.0 ± 68.3 µm; *p* = 0.003) (Figure 3D and Table 4).

However, the generalized linear model revealed that renal replacement therapy had no association with SFChT, although eGFR and phosphorus level had a similar association (Table 5).

### 3.5. Comparison of Both Eyes

Considering the correlation with renal function, we compared the mean SFChT of both eyes of each subject to investigate the similarity of both eyes at baseline. SFChT of both eyes of all subjects showed significant correlation even in patients in the pachychoroid and leptochoroid groups (Pearson coefficient 0.807, *p* < 0.001).

To investigate the effect of macular edema on SFChT, we compared edematous eyes with non-edematous contralateral eye. Of the 52 patients, 17 (32.7%) with unilateral DME had CRT ≥ 300 μm, whereas 28 (53%) of them had CRT ≥ 250 μm. Data analysis revealed that the eyes with edematous retina had more edematous choroids than contralateral non-edematous eyes (*p* = 0.001, *p* = 0.006) (Table 6).

## 4. Discussion

In this study, we investigated the correlation between renal function and ChT of the eyes of treatment-naïve PDR patients. This study revealed that the ChT of treatment-naïve PDR eyes was affected by eGFR and serum phosphorus level. Many previous studies have assessed the change in ChTs of the eyes of DR and DME patients; however, these studies have controversial findings [15,16,17,18]. The highly vascularized tissue characteristics of the choroid may have contributed to the confounding results. Thus, in this study, we attempted to control certain important confounding factors.

First, we included only treatment-naïve eyes. We previously reported that ChT decreases after PRP [15,16,17,18,19]. Other studies have also reported about the effect of PRP on ChT [20]. Moreover, several studies showed that ChT reduces after intravitreal injection of anti-VEGF [21]. Thus, we believed that it was essential to exclude eyes with treatment histories.

Second, we only included eyes with PDR. We previously reported that ChT decreases in the early stage of DR and significantly increases as the severity of DR increases [15]. A few studies have also described consistent findings of choroidal thickening in advanced DR [16]. However, some studies reported that ChT is reduced in the eyes of DM patients [18]. The controversial results of these previous studies suggest that DR progression could have diverse effects on ChT. Thus, to limit the effect of varying DR severity as a confounding factor and to clarify the correlation between renal function and ChT in this study, we only included treatment-naïve PDR patients.

With the control of these important confounding factors, the analysis of our study data produced several notable results. First, this study highlighted the correlation between renal function and ChT. eGFR and urine ACR have been widely used to monitor the renal function of CKD patients [22]. In the present study, eGFR was significantly lower in the leptochoroid group than in the pachychoroid group. Additionally, multivariate regression analysis showed a significant association between eGFR and SFChT. Moreover, patients with severely decreased eGFR had significantly less SFChT than patients with mildly or moderately decreased eGFR. Furthermore, the patients who underwent dialysis had significantly less SFChT than those who did not. These findings suggest a close correlation between SFChT and renal function of treatment-naïve PDR patients. Urine ACR was higher in the leptochoroid group than in the pachychoroid group; however, there was no significant difference in urine ACR values between the two groups. Moreover, regression analysis did not show a significant relationship between ChT and ACR. A few previous studies have reported inconsistent findings regarding the effect of microalbuminuria on the choroid; they reported a thinner or thicker choroid in patients with microalbuminuria than that in those without microalbuminuria [12,13]. In this study, we did not observe a correlation between albuminuria or ACR and ChT. We believe that the reversibility or improvement of albuminuria itself after medication may be responsible for the absence of a relationship between albuminuria and ChT because the use of angiotensin-converting enzyme inhibitors or angiotensin receptor blockers is recommended and widely prescribed to improve microalbuminuria and reduce the progression risk to macroalbuminuria [23].

Serum creatinine is one of the most widely used kidney biomarkers and is used in the estimated GFR (eGFR) equation [24]. However, serum creatinine levels can be affected by age, sex, race, diet, muscle mass, and use of medication [25]. Thus, eGFR using creatinine can sometimes be inaccurate. Cystatin C is a protein with a low molecular weight that acts as a cysteine protease inhibitor [26]. All nucleated cells produce cystatin C at a constant rate [26]. Thus, it is less affected by diet or muscle mass. Several studies suggest that cystatin C is a better marker of GFR than serum creatinine [26]. Furthermore, cystatin C-based eGFR is more accurate than creatinine-based equations. Therefore, we analyzed the relationship between ChT and GFR using cystatin C-based eGFR.

The results of this study also showed a close correlation between phosphorus level and SFChT. Decreased excretion of phosphorus owing to decreased renal function leads to hyperphosphatemia in CKD patients [27]. Phosphorus retention leads to high bone turnover and bone fragility and plays an important role in the development of vascular calcification and cardiovascular disease, as well as renal osteodystrophy [27]. Thus, hyperphosphatemia has been regarded as a prognostic marker for cardiovascular morbidity and mortality in CKD patients [27,28]. However, 79% (41/52) of participants in the present study had phosphorus levels within the normal range. Interestingly, higher phosphorus levels within the normal range are significantly associated with myocardial infarction or coronary death even in patients with normal kidney function [29]. Arteriolar sclerosis or vascular calcification owing to phosphorus retention may cause choroidal thinning in CKD patients.

In the present study, some treatment-naïve PDR patients had thicker choroids, whereas other patients had thinner choroids compared with the choroids of normal controls. This suggests that changes in the choroid of DR patients vary, as is noted in previous conflicting results [15,16,17,18]. This study suggests that this variation in choroidal changes is associated with renal function. Patients with decreased eGFR tended to have a smaller SFChT, whereas patients with elevated eGFR tended to have a larger SFChT. Elevation of GFR is known to precede a decline in renal function [29,30]. Interestingly, glomerular hyperfiltration is typically associated with glomerular hypertrophy and increased kidney size [31]. Thus, we cautiously suggest that the decrease in SFChT of DR patients is associated with a decline in renal function and the increase in SFChT is associated with glomerular hypertrophy.

This study showed that the choroid is closely associated with renal function. Compared with contralateral eyes, eyes with edematous retina had thicker choroids; thus, DME patients with declining renal function will have thinner choroids than healthy controls, if the control group does not comprise contralateral eyes but comprises healthy eyes. Therefore, the contralateral eyes of patients should be set as the control group when the effect of DME itself on the choroid is being investigated.

Some studies described thinning of the neuronal retina and thickening of choroidal layer in diabetic patients without diabetic retinopathy [32]. However, the participants have proliferative diabetic retinopathy and macular edema in this study. The different subject characteristics might lead to the differences in the results.

This study has several limitations. First, this was a retrospective cross-sectional study. Second, the number of enrolled patients was relatively small. However, we only included treatment-naïve PDR patients in this study. Generally, it is difficult to observe treatment-naïve PDR patients in the clinic or to enroll them in a study. Third, the number of DME patients in our study was limited. Another limitation of the present study is that the laboratory workup was conducted within a span of 4 weeks from ocular evaluations, which may have affected the results. Finally, renal replacement therapy could affect ChT in ESRD patients. These limitations should be addressed in future prospective studies. Despite these limitations, this is the first study to analyze the correlation between renal function and ChTs of DR patients.

In conclusion, SFChT is closely associated with renal dysfunction in treatment-naïve PDR patients. We believe that kidney function test and consultation for nephrology clinic should be considered in treatment-naïve PDR patient with choroidal thinning. Moreover, SFChT could be considered as a biomarker for renal function in patients with treatment-naïve PDR.

## Figures and Tables

**Table 1:** Baseline characteristics of the treatment-naïve proliferative diabetic retinopathy patients and the healthy controls.

Variables	Healthy Controls	Treatment-Naïve PDR Patients	*p*-Value ^†^
Number of patients	33	52	-
Number of eyes (R:L)	42 (19:23)	85 (44:41)	-
Number of eyes of patients with HTN, *n* (%)	0 (0%)	66 (77.6%)	-
Age, years	54.9 ± 15.1	52.9 ± 11.0	0.492
BMI (kg/m^2^)	25.1 ± 2.7	25.7 ± 4.2	0.461
BCVA, LogMAR	0.05 ± 0.07	0.24 ± 0.34	<0.001
Refractive error, SE	−1.0 ± 2.0	−1.1 ± 1.3	0.763
Intraocular pressure, mmHg	15.9 ± 2.9	15.9 ± 2.8	0.880
Central retinal thickness, µm	227.9 ± 26.3	268.3 ± 84.9	0.002
Mean retinal thickness, µm	281.5 ± 12.9	306.4 ± 60.8	0.007
Subfoveal ChT, µm	261.5 ± 21.2	316.0 ± 80.2	<0.001
Mean ChT, µm	215.9 ± 21.1	261.3 ± 64.1	<0.001

Values are number (%) or mean ± SD, unless otherwise indicated. ^†^ Independent *t*-test. BCVA: best-corrected visual acuity; BMI: body mass index; ChT: choroidal thickness; HTN: hypertension; L: left; LogMAR: logarithm of the minimum angle of resolution; PDR: proliferative diabetic retinopathy; R: right; SE: spherical equivalent.

**Table 2:** Comparison of the clinical characteristics and kidney parameters of diabetes mellitus patients with treatment-naïve proliferative diabetic retinopathy according to subfoveal choroidal thickness.

Variables	Pachychoroid Group	Leptochoroid Group	*p*-Value ^†^
Number of patients	30 (57.7%)	22 (42.3%)	
SFChT, µm	362.0 ± 45.8	236.8 ± 30.8	<0.001
BCVA, LogMAR	0.27 ± 0.38	0.30 ± 0.31	0.751
Intraocular pressure, mmHg	15.8 ± 2.7	15.1 ± 2.3	0.738
Refractive error (SE)	−0.7 ± 1.1	−1.0 ± 1.2	0.422
Number of HTN patients, *n* (%)	26 (53.1%)	11 (22.4%)	0.027 ^‡^
MAP, mmHg	97.2 ± 17.6	93.0 ± 12.4	0.369
Age, years	51.3 ± 10.5	55.5 ± 11.7	0.198
BMI (kg/m^2^)	26.1 ± 4.3	24.9 ± 4.1	0.362
Diabetes duration, years	12.1 ± 9.4	9.8 ± 6.8	0.367
FBS, mg/dL	169.2 ± 94.6	161.8 ± 107.7	0.801
HbA1c, %	8.3 ± 2.0	8.6 ± 2.8	0.568
ΔHbA1c ^§^, %	−1.1 ± 2.1	−1.1 ± 1.4	0.963
Creatinine, mg/dL	1.1 ± 0.8	1.8 ± 1.3	0.026
eGFR (Cystatin C), mL/min/1.73 m^2^	92.8 ± 34.3	38.4 ± 35.4	<0.001
Cystatin C, mg/L	1.2 ± 0.7	2.2 ± 1.2	0.004
Phosphorus, mg/dL	3.4 ± 0.5	4.2 ± 0.5	<0.001
Calcium, mg/dL	9.0 ± 0.4	8.9 ± 0.5	0.478
Urine albumin, mg/L	1440.3 ± 2918.3	1287.8 ± 2333.6	0.849
ACR, mg/g	832.1 ± 1278.7	1357.4 ± 2140.9	0.343

Values are number (%) or mean ± SD, unless otherwise indicated. ^†^ Independent *t*-test; ^‡^ Chi-square test; ^§^ Differences of HbA1c for 3–4 months; ACR: albumin/creatinine ratio; BMI: body mass index; BCVA: best corrected visual acuity; eGFR: estimated glomerular filtration rate; FBS: fasting blood sugar; HbA1c: glycated hemoglobin; HTN: hypertension; LogMAR: logarithm of the minimum angle of resolution; MAP: mean arterial pressure; SFChT: subfoveal choroidal thickness; SE: spherical equivalent.

**Table 3:** Univariate and multivariate linear regression analyses of the association between subfoveal choroidal thickness and chronic kidney disease parameters.

	Univariate			Multivariate		
	*B* ^†^	*Β* ^‡^	*p*-Value	*B* ^†^	*Β* ^‡^	*p*-Value ^§^
BCVA, LogMAR	−19.151	−0.092	0.530			
Intraocular pressure, mmHg	0.206	0.007	0.962			
Refractive error (SE)	5.391	0.084	0.566			
MAP, mmHg	0.913	0.196	0.178			
Age, years	−1.049	−0.150	0.361			
BMI (kg/m^2^)	1.375	0.079	0.592			
Diabetes duration, years	1.200	0.139	0.342			
FBS, mg/dL	0.122	0.164	0.261			
HbA1c, %	−6.991	−0.216	0.169			
ΔHbA1c ^‖^,%	4.097	0.104	0.476			
Creatinine, mg/dL	−13.050	−0.191	0.188			
eGFR (Cystatin C), mL/min/1.73 m^2^	1.123	0.591	<0.001	0.695	0.384	0.009
Cystatin C, mg/L	−28.804	−0.405	0.004	4.110	0.068	0.686
Phosphorus, mg/dL	−66.707	−0.565	<0.001	−46.895	−0.477	0.002
Calcium, mg/dL	19.839	0.116	0.464			
Urine albumin, mg/L	0.001	0.040	0.787			
ACR, mg/g	−0.005	−0.117	0.425			

^†^ Unstandardized (B) coefficient; ^‡^ Standardized (β) coefficient. ^§^ Enter multivariate linear regression analysis, adjusted *R*^2^ = 0.493. Variance inflation factors (VIF) were 2.213 (eGFR), 2.217 (phosphorus), and 2.859 (cystatin C). ^‖^ Differences of HbA1c for 3–4 months. ACR: albumin/creatinine ratio; BMI: body mass index; BCVA: best corrected visual acuity; eGFR: estimated glomerular filtration rate; FBS: fasting blood sugar; HbA1c: glycated hemoglobin; HTN: hypertension; MAP: mean arterial pressure; SE: spherical equivalent.

**Table 4:** Comparison of ocular parameters of treatment-naïve proliferative diabetic retinopathy patients who underwent hemodialysis and that of those that did not undergo hemodialysis.

Variables	Dialysis	No Dialysis	*p*-Value ^†^
Number of patients (%)	9 (17.3%)	43 (82.7%)	-
BCVA, LogMAR	0.44 ± 0.34	0.26 ± 0.35	0.160
Intraocular pressure, mmHg	14.8 ± 2.5	15.8 ± 2.5	0.280
Refractive error (SE)	−0.9 ± 0.9	−0.8 ± 1.2	0.807
Central retinal thickness, µm	274.6 ± 43.2	268.9 ± 89.3	0.853
Mean retinal thickness, µm	305.5 ± 38.0	305.8 ± 63.0	0.991
SFChT, µm	236.8 ± 95.9	320.0 ± 68.3	0.003
Mean ChT, µm	197.7 ± 71.5	266.3 ± 62.7	0.005

Values are number (%) or mean ± standard deviation, unless otherwise indicated. ^†^ Independent *t*-test. BCVA: best-corrected visual acuity; ChT: choroidal thickness; LogMAR: logarithm of minimum angle of resolution; PDR: proliferative diabetic retinopathy; SE: spherical equivalent; SFChT: subfoveal choroidal thickness.

**Table 5:** The generalized linear model between renal parameters and subfoveal choroidal thickness.

Variables	*B* ^*^	Standard Error	95% Confidence Interval	*p*-Value
Dialysis No	0 ^†^	–	–	–
Yes	−0.22	31.963	336.6–550.9	0.994
eGFR	0.667	0.2446	0.188–1.147	0.006
Cystatin	4.749	12.669	−20.084–29.581	0.708
Phosphorus	−50.119	13.951	−77.463–−22.775	<0.001

* Unstandardized (B) coefficient; ^†^ Set to zero because this parameter is redundant. eGFR: estimated glomerular filtration rate.

**Table 6:** Comparison of the central retinal thicknesses and subfoveal choroidal thicknesses of eyes with and without diabetic macular edema in patients with unilateral diabetic macular edema.

Variables	CRT < 300 µm	CRT ≥ 300 µm	*p*-Value ^†^
Eyes, *n*	17	17	
CRT, µm	242.6 ± 44.9	345.5 ± 135.0	0.004
SFChT, µm	286.5 ± 73.0	324.8 ± 87.0	0.001
Variables	CRT < 250 µm	CRT ≥ 250 µm	*p*-value ^†^
Eyes, *n*	28	28	
CRT, µm	235.0 ± 12.7	317.9 ± 102.4	<0.001
SFChT, µm	276.7 ± 63.2	300.4 ± 83.0	0.006

Values are number (%) or mean ± standard deviation, unless otherwise indicated. ^†^ Paired *t*-test; CRT: central retinal thickness; SFChT: subfoveal choroidal thickness.

## Supplementary Materials

The following are available online at https://www.mdpi.com/2077-0383/9/7/2171/s1, Table S1. The correlation between renal function parameters.
