# Supplementary material for: Strong Correlation of Renal Function with Choroidal Thickness in Patients with Type 2 Diabetes: Retrospective Cross-Sectional Study
Source: J Clin Med. 2020 Jul 9;9(7):2171. doi: 10.3390/jcm9072171 (PMC7408798; doi:10.3390/jcm9072171)
Supplement: Supplementary file 1 [file jcm-09-02171-s001.pdf]

Table S1. The correlation between renal function parameters.

		eGFR_CysC	ACR	Cystatin C	Phosphorus
eGFR_CysC	Pearson coefficients	1	-0.477	-0.727	-0.626
	P-value	–	0.001	<0.001	<0.001
ACR	Pearson coefficients		1	0.566	0.484
	P-value		–	<0.001	<0.001
Cystatin C	Pearson coefficients			1	0.727
	P-value			–	<0.001
Phosphorus	Pearson coefficients				1
	P-value				–

ACR, albumin/creatinine ratio; eGFR_CysC, estimated glomerular filtration rate based Cystatin C
